# Acceptability of HIV self-testing in African students: a cross-sectional survey in the Democratic Republic of Congo

**DOI:** 10.11604/pamj.2019.33.83.18586

**Published:** 2019-06-04

**Authors:** Serge Tonen-Wolyec, Francois-Xavier Mbopi-Kéou, Salomon Batina-Agasa, Ginette Claude Mireille Kalla, Michel Noubom, Ralph-Sydney Mboumba Bouassa, Jean De Dieu Longo, Jérémie Muwonga, Laurent Bélec

**Affiliations:** 1 Ecole Doctorale Régionale d’Afrique Centrale en Infectiologie Tropicale, Franceville, Gabon; 2 Faculty of Medicine, University of Bunia, Bunia, Democratic Republic of Congo; 3 Faculty of Medicine and Pharmacy, University of Kisangani, Kisangani, Democratic Republic of Congo; 4 University of Yaoundé I, Faculty of Medicine and Biomedical Sciences, Yaoundé, Cameroon; 5 The Institute for the Development of Africa (The-IDA), Yaoundé, Cameroon; 6 University of Dschang, Dschang, Cameroon; 7 Laboratory of Virology, Hôpital Européen Georges Pompidou and University of Paris Descartes, Paris, France; 8 Centre National de Référence des Maladies Sexuellement Transmissibles et de la Thérapie Antirétrovirale, Bangui, Central African Republic; 9 Unité de Recherches et d’Intervention sur les Maladies Sexuellement Transmissibles et le SIDA, Département de Santé Publique, Faculté des Sciences de la Santé de Bangui, Central African Republic; 10 National AIDS and STI Control Program’s Reference Laboratory, Kinshasa, Democratic Republic of Congo

**Keywords:** Self-testing, HIV, acceptability, supervised, unsupervised, Democratic Republic of Congo, Central Africa, French-speaking country

## Abstract

**Introduction:**

The empowerment of young people aged 15-24 years is a key component of an effective AIDS response. HIV self-testing (HIVST) is progressively being implemented in the Democratic Republic of Congo (DRC).

**Methods:**

Socio-demographic and behavioural factors associated with acceptability of HIVST were evaluated among university students in Bunia, DRC. A representative cross-sectional study was conducted using a self-administered semi-structured questionnaire.

**Results:**

A total of 1,012 students were recruited. Acceptability of unsupervised HIVST was higher in the group of young students as compared with older students and was markedly associated with prior knowledge on HIVST.

**Conclusion:**

Adapted communication about HIVST appears likely essential to increase the supply and use of HIVST among students in DRC.

## Introduction

The empowerment of young people aged 15-24 years is a key component of an effective AIDS response. High rate of youth in sub-Saharan Africa is unaware of its HIV status [1]. HIV self-testing (HIVST) can increase coverage of essential HIV services. Two HIVST approaches, supervised and unsupervised, have been proposed by WHO [2]. In unsupervised self-testing strategy, the self-tester performs the self-test on his own without any help and counselling and linkages are offered either by health care structures or communities or over the phone by trained counsellors or eventually through pharmacies [3]. However, in supervised self-testing, the self-tester may be assisted by healthcare or non-health care professional as counsellor to read the test results and provide counselling [3]. In the Democratic Republic of Congo (DRC), HIVST is being currently progressively implemented [4, 5]. Ituri is one of the eastern DRC post-conflict provinces with a high prevalence of HIV infection among young people because of the cross-border commercial activities with Uganda, sexual violence, child soldier phenomena and the use of young people in mining [6]. The aim of the study was to evaluate socio-demographic factors and behavioural factors associated with acceptability of supervised and unsupervised HIV self-testing among students from three major higher education institutions in the city of Bunia, capital city of the Ituri, DRC.

## Methods

From October to December 2018, a formative evaluation design using the quantitative study was employed to assess the acceptability of HIVST among students. A representative cross-sectional study was conducted using a self-administered semi-structured questionnaire. The survey was conducted in the *Université de Bunia (UNIBU)*, *Université Shalom de Bunia (USB)*, and *Institut Supérieur Pédagogique de Bunia (ISP/BUNIA)*. Inclusion criteria were age (≥18 years), to be regularly enrolled for the academic year 2016-2017, and to have willingness to provide verbal consent. Participants were trained for the instructions for use of a pilot immunochromatographic HIV self-test using typical pictures showing the principal steps of self-testing [5]. The questionnaire was redacted on the basis of (i) the different approaches and access to HIVST proposed by WHO (2); (ii) the literature review on the supervised and unsupervised HIVST [3]; and (iii) the literature review on the acceptability of the HIVST and the validation of the questionnaire among the students [7, 8]. The study was conducted following STROBE guidelines for reporting quantitative data [9]. The socio-demographic factors, the HIV counselling and testing practices (HCT) behaviours and the sexual behaviours were addressed according to the following topics: sex, age group, partnership and civil status, educational level, ever tested for HIV, sexual activity, number of sex partners in past six months, knowledge about HIV self-testing.

To assess the opinions on HIVST, students were asked if they were aware of HIVST, whether they would use HIVST as a testing option, their reaction when HIVST is reactive (positive), and their reaction when HIVST is nonreactive (negative) and their opinion on the results of self-testing of sexual partners. To assess their preferences, students were asked about their preference for self-test access, their willingness of substitution in which users replace HCT with self-testing, their cost preference and their willingness to pay the HIVST, and their preference for counselling. Ethical clearance for this study was obtained from Ethics Committee of the School of Public Health of Kinshasa's University. All participants gave their verbal informed consent to participate in this study. All data were entered into an Excel file and analyzed on SPSS 20.0 (Chicago, IL). Means ± standard deviations were calculated for quantitative variables and proportions for categorical variables. The Pearson's χ^2^test or Fisher's exact test were used for comparison of the frequencies. The strength of statistical associations was measured by crude and adjusted odds ratios (OR) and their 95% confidence intervals. The P-value < 0.05 was considered as statistically significant. Missing values were replaced by the single imputation using regression method.

## Results

A total of 1,080 participants were recruited. Of those 68 students were excluded because they did not give their consent. Finally, 1,012 students participated in the survey, including 34.1% (n = 345) from UNIBU, 33.8% (n = 342) from USB, and 32.1% (n = 325) from ISP/BUNIA; and equal proportion of male (50.1%) and females (49.9%). More than three-quarters (84.0%) of participants were single. The mean age of the students was 23 ± 4 years; the majority (70.7%; n = 715) were young students (age-24 years). More than half (53.7%; n = 543) had yet received HIV counselling and testing practices in the past. The majority (88.8%; n = 899) reported previous sexual intercourse; among those, 18.7% (n = 168) had simultaneously (11.3%; n = 102) or serially (7.3%; n = 66) multiple sexual intercourses in previous six months. A minority (13.2%; n = 119) consistently used condoms. Note that, all participants were heterosexual. Data show that only 6% (n = 54) reported that they knew the HIV status of their sexual partners. Less than half of participants (45.5%; n = 460) reported being already informed about the HIV self-testing. Overall, the acceptability of HIVST as a testing option was 81.4% (n = 824). The willingness to see the doctor for confirmation and care after a reactive HIVST was high among old (age > 24 years) students (86.9%; n = 258) than young (80.7%; n = 577; P = 0.02). Concerning the rate of substitution, 40.4% (n = 409) of participants agreed to take the place of HCT for self-testing strategy. The majority (77.9%; n = 557) of young students reported that post-test counselling should be essential for use of HIV self-testing. Regarding the willingness to buy HIV self-test, 58.4% (n = 591) of students had a favourable opinion to buy it, and the mean of price was 3 US $ (range, 0-22). In bivariate analysis, the variables significantly associated with the acceptability of supervised and unsupervised HIVST were the following: “sex”, “age group”, “partnership and civil status”, “educational level”, “past-history of HIV testing”, “sexual activity”, “number of sex partners in the past six months” and “knowledge about HIVST.” Results of logistic regression analyses are depicted in Table 1. The acceptability of unsupervised HIVST was higher in the group of young students as compared with older students (crude OR: 3.4 [95% CI: 2.6-4.6]; adjusted OR: 3.6 [95% CI: 2.4-5.4]; P < 0.0001); the acceptability of unsupervised HIV self-testing was significantly associated with prior knowledge on HIVST (crude OR: 2.9 [95% CI: 2.2-4.0]; adjusted OR: 2.8 [95% CI: 2.0-3.8]; P < 0.0001). Finally, partnership and civil status and past-history of HIV testing were associated with acceptability of unsupervised HIVST with logistic regression.

**Table 1:** Factors associated with the acceptability of supervised and unsupervised self-testing in 1,012 students from three major higher education institutions in the city of Bunia, Democratic Republic of the Congo

Characteristic	Acceptability of supervised HIV self-testing					Acceptability of unsupervised HIV self-testing					Total *n =1,012*
	*n* (%)	cOR [95% CI]	P*	aOR [95% CI]	P**	*n* (%)	cOR [95% CI]	P*	aOR [95% CI]	P**	
**Sex**											
Male	298 (58.8)	0.9 [0.7–1.3]	0.991	0.8 [0.6–1.0]	0.061	353 (69.6)	0.7 [0.5–0.9]	0.006	0.8 [0.6–1.2]	0.269	507
Female	297 (58.8)	Reference		Reference		390 (77.2)	Reference		Reference		505
**Age group**											
Younger ≤ 24	383 (53.6)	0.5 [0.3–0.6]	< 0.0001	0.7 [0.5–1.0]	0.077	579 (81.0)	3.4 [2.6–4.6]	< 0.0001	3.6 [2.4–5.4]	< 0.0001	715
Older > 24	212 (71.4)	Reference		Reference		164 (55.2)	Reference		Reference		297
**Partnership and civil status**											
Single	461 (54.2)	0.1 [0.01–0.5]	< 0.0001	0.1 [0.01–0.6]	0.013	641 (75.4)	7.8 [2.1–13.6]	< 0.0001	5.7 [1.6–20.8]	0.008	850
Married/partnered	114 (80.9)	Reference		Reference		84 (59.6)	Reference		Reference		141
Other	20 (95.2)	6.2 [1.3–0.3]		4.7 [0.6–36.6]	0.143	18 (85.7)	8.1 [2.6–24.2]		5.9 [1.6–21.5]	0.008	21
**Educational level**											
Undergraduate	380 (54.3)	Reference	< 0.0001	Reference		548 (78.3)	Reference	< 0.0001	Reference		700
Graduate degree	215 (68.9)	1.9 [1.4–2.5]		1.3 [0.9–1.8]	0.157	195 (62.5)	0.5 [0.3–0.6]		1.0 [0.7 – 1.5]	0.930	312
**Ever tested for HIV**											
Yes	295 (54.3)	Reference	0.002	Reference		400 (73.7)	Reference	0.849	Reference		543
Non	300 (64.0)	0.7 [0.5–0.9]		0.7 [0.5–1.1]	0.084	343 (73.1)	1.0 [0.8–1.4]		1.8 [1.0–3.1]	0.05	469
**Sexually active**											
Yes	538 (59.8)	1.4 [0.9 – 2.1]	0.056	1.1 [0.7–1.7]	0.728	655 (72.9)	0.8 [0.5–1.2]	0.225	0.7 [0.4–1.3]	0.286	899
No	57 (50.4)	Reference		Reference		88 (77.9)	Reference		Reference		113
**Number of sex partners in past six months**											
None	219 (52.3)	2.0 [1.0–2.9]	0.001	1.2 [0.7–2.0]	0.591	310 (74.0)	1.3 [0.7–2.3]	0.073	1.5 [0.8–2.8]	0.256	306
One partner	279 (65.6)	0.6 [0.4–0.9]		0.8 [0.5–1.4]	0.449	316 (74.4)	1.1 [0.6–1.9]		0.9 [0.5–1.7]	0.786	425
Two partners	49 (54.4)	2.1 [0.8–3.2]		1.4 [0.7–2.7]	0.292	56 (62.2)	0.5 [0.3–4.5]		0.6 [0.7–3.3]	0.238	90
More than two partners	48 (61.5)	Reference		Reference		61 (78.2)	Reference		Reference		78
**Knowledge about HIV self-testing**											
Yes	280 (60.9)	1.2 [0.9 – 1.5]	0.221	[0.9–1.7]	0.55	387 (84.1)	2.9 [2.2–4.0]	< 0.0001	2.8 [2.0–3.8]	< 0.0001	460
No	315 (57.1)	Reference		Reference		356 (64.5)	Reference		Reference		552

* P-value calculated using Pearson’s χ2 test or Fisher's exact test;

** P-value calculated using regression analysis;

aOR: adjusted Odds ratios; cOR: crude Odds ratios; CI: confidence interval; *P*: *P*-value

## Discussion

We herein evaluated the acceptability of HIVST in students in Bunia, Democratic Republic of Congo. Our observations show that the unsupervised HIVST approach is much more acceptable (81%) among young students (age ≤ 24 years) and that the acceptability of unsupervised HIV self-testing is associated with prior knowledge on HIVST. These findings are reminiscent to previous studies in South Africa on the acceptability of HIVST among young and adolescents reporting high rates of acceptability, generally greater than 80% [10]. The understanding of supervised and unsupervised self-testing strategies was previously evaluated by Pant Pai and colleagues in order to guide effective implementation of HIVST in high-income and resource constrained settings [3]. Those authors evaluated the feasibility to implement unsupervised oral fluid-based self-testing strategy which appears effective despite variable accuracy estimates obtained by self-testers [3]. Nonetheless, our previous observations indicate a good practicability and performance of finger-stick whole-blood HIVST among highly educated people, demonstrating the potential of using an unsupervised HIVST [5, 11]. Thus, the implementation of unsupervised finger-stick whole blood-based self-testing strategy appears effective in young students because of the higher acceptability as demonstrated by our finding. Finally, the majority (77.9%) of the study’s young students reported that post-test counselling should be essential for use of HIV self-testing. These observations confirm that post-test counselling should be accessible to people who conduct HIVST even in sub-Saharan Africa [3, 10, 11].

## Conclusion

In conclusion, our study demonstrates high acceptability of unsupervised HIVST among young students in association with prior knowledge of HIVST and possibility of post-test counselling. Adapted communication about HIVST appears likely essential to increase the supply and use of HIVST among students in DRC.

### What is known about this topic

- HIV self-testing (HIVST) is a novel strategy that can enable early HIV testing, prevention interventions and care in key populations at high risk of HIV infection, such as young people aged 15-24 years;
- In the Democratic Republic of Congo (DRC), HIVST is being currently progressively implemented;
- The supervised and unsupervised HIVST are the two approaches of self-test kits distribution as proposed by World Health Organization (WHO) in 2013 to implement HIV self-testing.

### What this study adds

- This study demonstrates high acceptability of unsupervised HIVST rather than supervised HIVST among people aged 18-24 years in association with prior knowledge of HIVST and possibility of post-test counselling;
- Adapted communication about HIVST appears likely essential to increase the supply and use of HIVST among students in DRC.

## Competing interests

The authors declare no competing interests.
